# Phenolic Profile by HPLC-PDA-MS of Greek Chamomile Populations and Commercial Varieties and Their Antioxidant Activity

**DOI:** 10.3390/foods10102345

**Published:** 2021-10-01

**Authors:** Nektaria Tsivelika, Maria Irakli, Athanasios Mavromatis, Paschalina Chatzopoulou, Anastasia Karioti

**Affiliations:** 1 Laboratory of Genetics and Plant Breeding, Department of Agriculture, Faculty of Agriculture Forestry and Natural Environment, Aristotle University of Thessaloniki, 54124 Thessaloniki, Greece; riatsivel@gmail.com (N.T.); amavromat@agro.auth.gr (A.M.); 2 Hellenic Agricultural Organization—DEMETER, Institute of Breeding and Plant Genetic Resources, Thermi, 57001 Thessaloniki, Greece; irakli@ipgrb.gr (M.I.); chatzopoulou@ipgrb.gr (P.C.); 3 Laboratory of Pharmacognosy, School of Pharmacy, Aristotle University of Thessaloniki, University Campus, 54124 Thessaloniki, Greece

**Keywords:** *Matricaria recutita*, Greek chamomile, phenolics, apigenin, HPLC-PDA-MS, antioxidant activity

## Abstract

The phenolic profile of Greek chamomile populations was investigated by HPLC-PDA-MS. For comparison, three commercial varieties (Banatska, Lutea and Goral) cultivated under the same conditions were included in the study. All samples exhibited similar qualitative patterns but differed in their quantitative characteristics. Overall, 29 constituents were detected, belonging to phenolic acids, flavonol glycosides, flavone glycosides (mainly apigenin derivatives) and acylated polyamines. Quantitative results showed that both Greek populations had a high content in apigenin derivatives (0.39 and 0.31 %*w/w*) and caffeoylquinic acids (0.96 and 0.81 %*w/w*), whereas they had the highest amount of flavonol glycosides among the tested samples. Greek populations were comparable to the Banatska variety, while they were superior to the Lutea and Goral varieties cultivated under the same conditions. Results demonstrate that Greek chamomile populations studied here, are an excellent source of a wide range of phenolics which contribute to the medicinal and antioxidant properties of this herbal remedy. Antioxidant tests showed that chamomile extracts from the studied materials, especially from the Greek populations possess antioxidant activity, corresponding to their polyphenol content. This is the first report on the phenolic constituents of *Matricaria recutita* growing in Greece and well-established chamomile varieties.

## 1. Introduction

*Chamomile (Matricaria recutita* L. Rauschert., syn. *M. chamomilla* L.) is one of the most important, widely used aromatic and medicinal plant. It has been used as a natural remedy since antiquity, for its anti-inflammatory, antiseptic, analgesic, antimicrobial, antispasmodic and sedative properties [1]. Chamomile dried flower heads are used in the form of herbal teas or hydroalcoholic extracts to relieve symptoms and inflammatory conditions of the gastrointestinal track, including bloating and spasms, minor ulcers and inflammations of the mouth, throat, skin and superficial wounds irritations of skin and mucosae [2,3]. As a dietary supplement chamomile is widely consumed in everyday life as anxiolytic and gentle sleep aid [4]. Moreover, chamomile has been proposed as functional food as it can potentially relieve the metabolic overload effects of high sugar diets [5]. Functional foods are considered processed foods having disease-preventing and/or health-promoting benefits only if they are consumed regularly in an adequate measure [6]. In this view, chamomile has been suggested to prevent osteoporosis, osteoarthritis, etc [7,8,9]. Recently, chamomile decoctions were incorporated into cottage cheese improving its antioxidant potential and increasing the product’s shelf life [10]. 

Dietary supplements are a continuously growing market and have become increasingly popular, especially under the notion that “everything natural is healthy”. While these products initially were launched as vitamins and minerals [10], with the aim to correct nutritional deficiencies, they have expanded to herbal extracts. In Europe food supplements are subject to food law and are under the authorization of EFSA (European Food Safety Authority). As a result, their legislation is not so strict as the one of the medicinal products, allowing them to be marketed via pharmacies, herbal shops, health food-stores, super-markets, internet, etc., which has contributed further to their wide availability and popularity. The unreasonable use by the consumers along with the fact that food supplements may cause undesirable effects especially if consumed by sensitive populations (infants, babies, pregnant women, aged, allergic, patients under heavy medication) has often raised health concern about these borderline products and their free use [11,12,13]. In this context, chamomile may be considered a relatively safe plant. With the exception of allergies due to its pollen, it is well tolerated and its longstanding use, since antiquity, guarantees its efficacy and safety [14].

The medicinal and health benefit properties of chamomile are associated to its bioactive compounds, mainly to the essential oil (consisting predominately of the sesquiterpenes α-bisabolol, bisabolol oxides A and B and chamazulene), and to substances mainly present in the aqueous extract (referring mostly to phenolic acids, flavonoids and coumarins) [15]. Herniarin and umbelliferone (coumarins), chlorogenic acid and caffeic acid (phenylpropanoids), apigenin, apigenin-7-O-glucoside, luteolin and luteolin-7-O-glucoside (flavones), quercetin and rutin (flavonols) are reported as the main phenolic compounds [6]. In chamomile decoctions and extracts most of the flavonoids, among them apigenin, occur as glycosides and are highly stable and water-soluble [1,16,17]. The solubility of phenolics and flavonoids in water makes chamomile tea a rich source of healthy ingredients and antioxidant compounds. Indeed, several studies have been reported on the health promoting, anti-inflammatory, cytostatic and anticancer properties of apigenin and its glycosidic derivatives, indicating their safe use [1,18,19]. Apigenin and its glycosides are considered a suitable marker for chamomile quality and safety [20]. These quality characteristics are recognized by the European Pharmacopoeia regulations according to which chamomile flowers should contain 4 mL/kg of blue essential oil and at least 0.25% of apigenin-7-glucoside (*w/w*, dried herbal drug) [21]. European Pharmacopeia proposes a method including treatment of the herbal drug with ammonia leading to alkaline hydrolysis of apigenin acylated glycosides and determination of apigenin-7-O-glucoside. Although it is a fast and practical method, aiming to the determination of all apigenin derivatives, it causes degradation of many other components, thus altering considerably the phenolic profile of the extracts [22]. A literature survey showed that most of the quality assessment studies on chamomile focus mainly on the essential oils [23,24,25,26], whereas the majority of HPLC analytical methods limit to the determination of apigenin and apigenin-7-O-glucoside in extracts or decoctions, without measuring the flavonoid/phenol content in the herbal drug, which is nevertheless the raw material. The only exception is the report on the qualitative and quantitative profile of phenolic constituents of several commercial chamomile samples by Avula et al. [27]. Unfortunately, in this report, the chamomile varieties under investigation, are not defined, beyond the fact that there are no data concerning the flavonol content. Although an HPLC-PDA-TOF-MS was used, the chromatographic profile focused mainly on the main compounds as well as minor constituents were not considered.

The present study aimed to address all the above points. Five accessions of cultivated chamomile were considered, and their phenolic profile was assessed by HPLC-PDA-MS. The chamomile samples consisted of flowers of two Greek cultivated chamomile populations (Figures S1 and S2) along with three commercial varieties (V3—Banatska (diploid) and two tetraploids V4 -Lutea and V5- Goral). With the exception of Goral, for the other two varieties, although they are widely cultivated, there are no data available in the literature concerning their chemical content. It is also the first time that is shed light to phenolic profile of Greek chamomile. This is of great importance if we take into consideration the climatic conditions of Greece which favor plants of the Asteraceae family [28]. Cultivation of all five populations and chamomile varieties was conducted under the same conditions in the experimental field of Institute of Breeding and Plant Genetic Resources (PB&GRI) in order to allow for a comparison between the different samples. Furthermore, an improved extraction scheme was applied, able to extract a wider variety of phenolic constituents in order to have as a more complex metabolic fingerprint as possible. HPLC-PDA-MS was used to characterize qualitatively and quantitatively chamomile accessions under investigation. This is the first report on the chemical profile of Greek chamomile, as well as of the reported commercial chamomile varieties.

## 2. Materials and Methods

Plant material consisted of chamomile flowers was collected from two Greek populations: Populations S1 and S2, preserved in the ex-situ field collection of PB&GRI (Thessaloniki—Greece) [26]. Germplasm of S1 originated from a native to North Greece chamomile population [26], and S2 was derived from population S1, after continuous selection of superior plants, for three consecutive years, following appropriate breeding methods (unpublished data). Moreover, for comparison reasons, the phenolic profile of three European chamomile varieties was examined: flowers were collected from a diploid chamomile variety Banatska (V3) and from two tetraploid commercial varieties Lutea (V4) and Goral (V5). Plants of S1, S2, V3, V4 and V5 were cultivated under the same environmental conditions in the experimental field of PB&GRI (Thessaloniki—Greece) and received the same cultivation treatments. In particular, all the accessions were grown under ‘organic green’ farming conditions, without fertilization or pesticide inputs. The climatic conditions at the experimentation area are characterized from mild winter, warm spring and summer with high relative humidity. The soil properties were as follows: soil type: red loam, pH: 8.15%, EC: 0.426 mS cm^−1^, organic matter: 1.19%, CaCO_3_: 4.8%, NO_3_: 13.5 ppm, P: 4.41 ppm, K: 194 ppm, Fe: 7.76 ppm, Zn: 0.62 ppm, Mn: 6.41 ppm, Cu: 1.34 ppm. Chamomile flowers were collected from all accessions at full flowering, and after collection, they were dried in well ventilated, dark room, at ambient temperature.

### 2.1. Sample Preparation of Phenolic Extracts

Dried chamomile flowers were collected from at least ten plants of each chamomile population/variety. Ten grams of each sample (population and/or variety) of *M. recutita* dried flowers, grounded and mixed to obtain a homogenous sample. Approximately 200 mg of sample, were accurately weighed and ultrasonicated twice with 10mL methanol 100% for 10 min and then twice with methanol:water 70:30. The extracts were filtered through paper and the filtrates were combined and adjusted to 100.0 mL using methanol 100%. The solutions were further diluted (1:2), filtered through Nylon filters (0.45 µm pore size) and immediately injected. The extractions of all samples were performed in triplicate and injected three times (45 injections total).

### 2.2. Chemicals

All solvents were purchased from Sigma-Aldrich (Taufkirchen, Germany). Water was purified by a Milli-Qplus system from Millipore (Milford, MA, USA). Sephadex LH-20 was purchased from Sigma-Aldrich. Nylon filters (0.45 µm pore size) were from Agilent (Agilent Technologies, Palo Alto, CA, USA).

### 2.3. Standards

Apigenin-7-O-glucoside was purchased form Extrasynthèse (Genay Cedex, France). Rutin (95% purity) and chlorogenic acid (95% purity) were purchased from Sigma Aldrich (Taufkirchen, Germany). A series from stock solutions were prepared and kept at −20 °C. From these stock solutions a series of fresh working solutions were prepared by diluting them in methanol 100%.

### 2.4. Determination of Total Phenolic and Total Flavonoid Contents

*Total phenolic content (TPC)* was determined according to the Folin Ciocalteu method [29], slightly modified by Irakli et al. [30]. Briefly, 0.2 mL of chamomile phenolic extracts, 0.8 mL of diluted Folin–Ciocalteu reagent (1:10, *v/v*), 2 mL sodium carbonate solution 7.5% and 7 mL distilled water were mixed. The absorbance of mixture was recorded at 725 nm after 60 min incubation in a dark place. TPC assays was carried out triplicate and the results were expressed as mg of gallic acid equivalents (GAE) per g of chamomile extract (mg GAE/g extract).

*Total flavonoid content (TFC)* was assessed by using the colorimetric method of aluminum chloride of Bao et al. (2005) [31] with minor modifications. Briefly, 0.2 mL of diluted chamomile phenolic extracts were mixed with 0.15 mL 5% NaNO_2_, 0.15 mL 10% AlCl_3_∙6H_2_O and 0.5 mL 1M NaOH; the absorbance was recorded at 510 nm after 30 min incubation. TFC determination was executed in triplicate and the results were expressed as milligrams of catechin equivalents per g of chamomile extracts (mg CATE/g extract).

### 2.5. Determination of Antioxidant Activity by DPPH Assay

*DPPH radical scavenging activity (RSA-DPPH)* of chamomile extracts was determined according to Yen and Chen [32] with some modifications. A solute 2.85 mL of 0.1 mM DPPH (2,2-diphenyl-1-picrylhydrazyl) in methanol was mixed with 100 μL diluted chamomile methanolic extracts and the decrease in absorbance was measured at 516 nm after 5 min. The calibration curve was performed with Trolox solutions at different concentrations (100–1000 μM). The analyses were carried out in triplicate and the results were expressed as mg Trolox equivalents (TE) per g chamomile extract (mg TE/g extract).

### 2.6. Determination of Antioxidant Activity by ABTS Assay

*ABTS radical scavenging activity* was assessed in chamomile extracts by using the 2,2’-azinobis (3-ethylbenzothiazoline-6-sulfonic acid) (ABTS) reagent according to the protocol of Re et al. (1999) [33], as adopted by Irakli et al. [30]. Briefly, ABTS+ solution was prepared by mixing 7.4 mM ABTS and 2.6 mM potassium persulfate in equal volumes and adjusted its absorbance of 0.70 ± 0.02 at 734 nm. 3.9 mL of the above ABTS+ solution was added to 0.1 mL of diluted phenolic extract and the absorbance at 734 nm was recorded after 4 min against a blank. The results were expressed as mg Trolox equivalents per g of chamomile extract (mg TE/g extract).

### 2.7. HPLC-PDA-MS Analysis Instrumentation

Analysis was carried out using an LC-PDA-MS Thermo Finnigan system (LC Pump Plus, Autosampler, Surveyor PDA Plus Detector) interfaced with an ESI MSQ Plus (Thermo Finnigan) and equipped with an Xcalibur software. The same column, timetable and flow rate were used during the HPLC-MS analyses. The mass spectrometer operated in both negative and positive ionization modes, scan spectra were from *m*/*z* 100 to 1000, gas temperature was at 350 °C, nitrogen flow rate at 10 L/min, and capillary voltage 3200 V. The cone voltage was in the range of 60–100 V. The column was a SB-Aq (Agilent) RP-C18 column (150 mm × 3 mm) with a particle size of 5 µm maintained at 30°C. The eluents were H_2_O at pH 2.8 by formic acid (0.05% *v/v*) (A) and acetonitrile (B) and with a flow rate of 0.4 mL/min. Gradient program was as follows: 0–15 min, 85–79% A; 15–25 min 79–77% A; 25–45min, 77%–65% A; 45–53 min, 65%–35% A; 53–56 min, 35%–85% A; 56–60 min, 85% A. Injected volume of the samples was 5 μL of solution. The UV–vis spectra were recorded between 220 and 600 nm and the chromatographic profiles were registered at 315, 330 and 350 nm.

### 2.8. Qualitative and Quantitative Determination of Flavonoids and Caffeoyl Quinic Acids in the Herbal Drug

The identification of the constituents was performed by HPLC-PDA and MS analysis by comparing the retention time, the UV and MS spectra of the peaks in the samples with those of authentic reference samples or isolated compounds [34] and, in some cases, data reported in the literature. For the quantitative determination of flavonoids, the method of external standard was applied, using apigenin-7-O-glucoside and rutin as standard. For caffeoylquinic acid derivatives chlorogenic acid was used as external standard. The regression curve was obtained by measuring each point in triplicate. Measurements were performed at 330 nm for apigenin and caffeoylquinic acid derivatives and at 350 nm for flavonols. Results were adjusted using a molecular weight correction factor and are expressed as % mg of dried herbal drug, according to the requirements of the European Pharmacopoeia and the European Medicines Agency.

### 2.9. Statistical Analysis

Greek chamomile populations (S1, S2) and varieties (V3, V4 and V5) were quantified for their main phenolic compounds, i.e., apigenin derivatives, flavonol and dicaffeoylquinic acid derivatives, and their total phenolic content, total flavonoid content, and antioxidant activity (assessed by DPPH and ABTS) were estimated as well. The samples were analyzed in triplicate. The results are expressed as the means and the data were analyzed with analysis of variance (ANOVA), through the statistical package SPSS ver. 22. The Duncan’ s multiple range test was used for mean comparisons and the statistical significance was determined at α = 0.05.

## 3. Results

**Figure 1:**
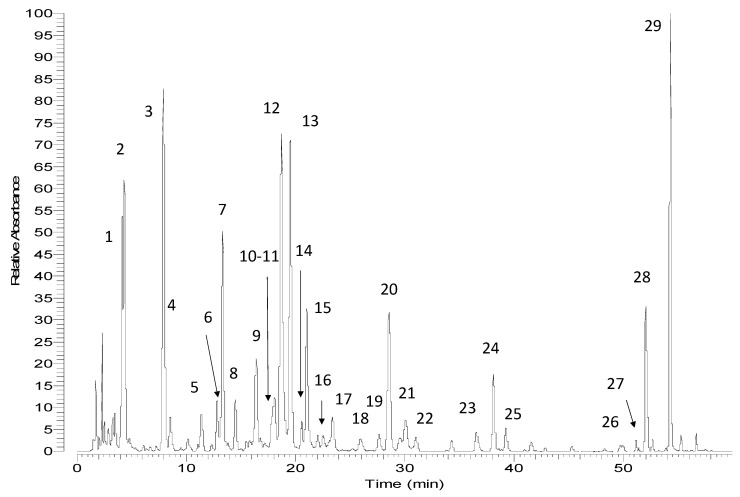
Representative chromatograms of *Matricaria recutita* flos from Greece. Experimental conditions: column: Zorbax SBAq RP-C18 (150 × 3.0 mm), particle size 5µm (Agilent) at 30 °C. **1** Chlorogenic acid; **2** cis-2-hydroxy-4-methoxycinnamic-oxo-2-O-β-D-glucopyranoside; **3** quercetagenin-3-O-glucoside; **4** trans-2-hydroxy-4-methoxycinnamic-oxo-2-O-β-D-glucopyranoside; **5** quercetin-7-O-hexoside; **6** patuletin-7-O-glucoside; **7** patuletin-3-O-glucoside; **8** luteolin-7-O-glucoside; **9** dicaffeoylquinic acid derivative; **10** isorhamnetin-7-O-hexoside; **11** isorhamnetin-3-O-glucoside; **12** 3,5-O-dicaffeoylquinic acid; **13** apigenin-7-O-glucoside; **14** chrysoeriol-7-O-glucoside; **15** 4,5-O-dicaffeoylquinic acid; **16** Isorhamnetin-3-O-malonylhexoside tentatively; **17** acylated derivative of cis-2-hydroxy-4-methoxycinnamic-oxo-2-O-β-D-glucopyranoside; **18** Isorhamnetin-7-O-malonylhexoside tentatively; **19** apigenin-7-O-malonylhexoside **20** apigenin-7-O-malonylhexoside + apigenin-7-O-acetylhexoside; **21** apigenin-7-acetylhexoside; **22** acylated derivative of trans-2-hydroxy-4-methoxycinnamic-oxo-2-O-β-D-glucopyranoside; **23** apigenin-7-acetylhexoside isomer; **24** Apigenin-7-O-acetylmalonyl-hexoside; **25** Apigenin-7-O-acetylmalonyl-hexoside; **26** N1(Z)-N5(Z)-N10(Z)-N14(Z)-tetra-p-coumaroyl spermine/thermospermine (cis-isomers); **27** N1(Z)-N5(Z)-N9(Z)-N14(Z)- tetra-p-coumaroyl spermine/thermospermine (cis and trans-isomers); **28** N1(E)-N5(E)-N10(E)-N14(E)- tetra-p-coumaroyl spermine (trans-isomer); **29** N1(E)-N5(E)-N9(E)-N14(E)-tetra-p-coumaroyl thermospermine (trans-isomer).

**Figure 2:**
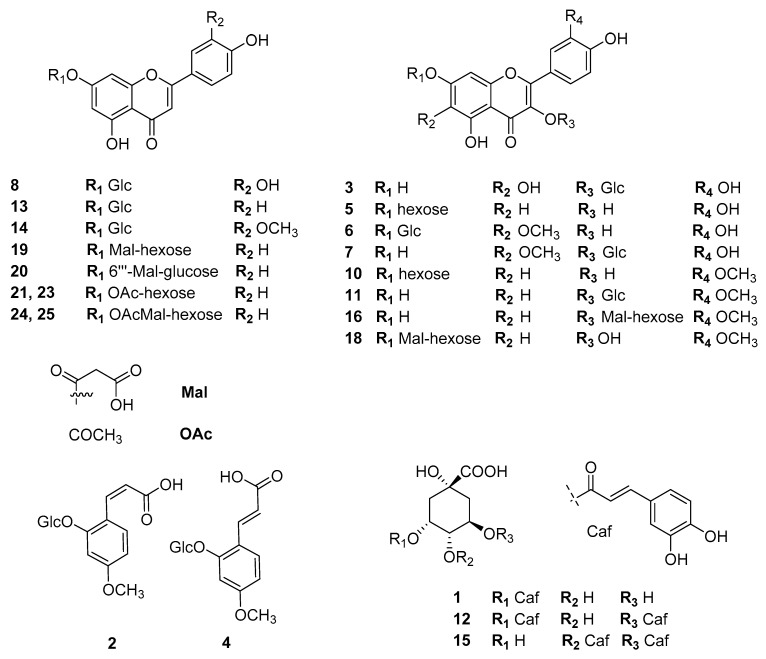
Flavonoids and phenolics detected in *Matricaria recutita* flowers.

**Figure 3:**
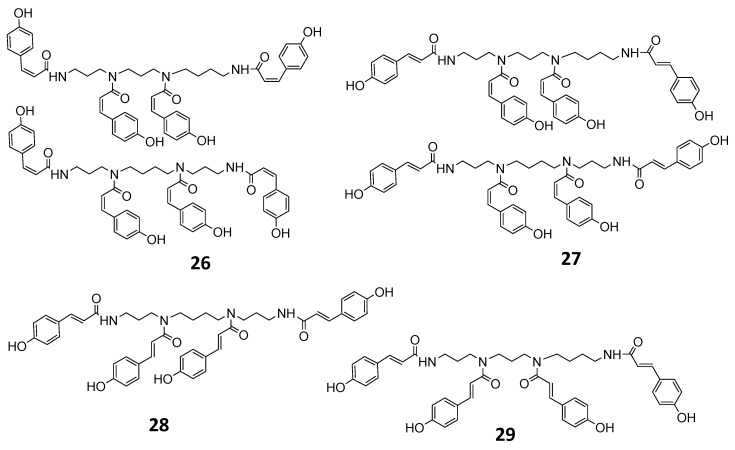
Acylated polyamines detected in *Matricaria recutita* flowers.

The (poly) phenolic profile of two Greek chamomile populations along with three commercial varieties was evaluated qualitatively and quantitatively. The constituents of the hydromethanolic extracts were identified by UV and MS spectral data and the qualitative profiles of all samples were quite similar. In Figure 1 a representative HPLC-PDA chromatogram of the investigated *M. recutita* extracts at 330nm is shown. Chromatograms of all the studied extracts are given in the Supplementary Data (Figures S1 and S2). Data concerning identification of the peaks are shown in Table 1, where the retention time, UV–vis absorptions and electrospray ionization mass spectrometry in both positive and negative ion mode of all the compounds detected in the chamomile extracts are reported. The analytical system led to the separation and identification of the majority of the constituents. Overall, twenty-nine compounds were detected and identified (Figure 2 and Figure 3), belonging to four representing classes of compounds: (i) phenolic acids and conjugates; (ii) flavonol glycosides; (iii) flavone glycosides and (iv) polyamine conjugates. In general, negative ionization mode yielded many diagnostic fragments, while positive ionization mode was used as confirmation when needed.

### 3.1. Identification of Phenolic Acids and Conjugates

The first group of peaks of all the chromatograms consisted of three peaks at 4.17, 4.37 and 7.88 min which showed UV spectra typical of the presence of phenolic derivatives. Especially peak **1** with maximum absorbance at 326 nm and a shoulder around 296 nm, was characteristic of a caffeoylquinic acid derivative. Pseudomolecular ions at 353 [M−H]^−^ and the characteristic fragment ion at *m/z* = 191 (Table 1), suggested the presence of a chlorogenic acid derivative. Indeed, compound **1** was identified as chlorogenic acid (5-caffeoylquinic acid) by comparing its UV spectra and retention times with those of the reference substance. Peaks at 4.37 and 7.88 min had characteristic absorbances of cis (301 nm) and trans (318 nm) cinnamic acid moieties, respectively. Peaks **2** and **4** were identified as cis- and trans-2-hydroxy-4-methoxycinnamic-oxo-2-O-β-D-glucopyranoside. The fragment at *m/z* = 193 corresponded to the aglycone group and its difference by 162 amu from the pseudomolecular ion (*m/z* =355) suggested the neutral loss of a sugar moiety [M-hexose-H]^−^. A successive neutral loss of CO_2_ corresponded to the detachment of a carboxyl group *m/z* = 149 [M-hexose-CO_2_-H]^−^, indicating that in the cinnamic group the carboxylic acid was free, non-esterified. Further loss of a methyl group was observed at *m/z* = 134 [M-hexose-CO_2_-CH_3_-H]^−^ and was typical of an homolytic cleavage of a methyl belonging to a methoxy group [35]. Finally, adduct ions at *m/z* 711 [2M-H]^−^ and 379.0 [M+Na]^+^ confirmed the structure. Both compounds were unambiguously identified on the basis of co-elution with the respective compounds recently isolated in our lab [34]. Three more peaks at 16.37, 18.70 and 21.03 min were attributed to di-caffeoylquinic acid derivatives. All of them gave common pseudomolecular ions at m/z 515 [M−H]^−^, and fragments at *m/z* = 353 [M−162-H]^−^ and 191 [M−162–162–H]^−^, suggesting the neutral loss of two caffeoyl units and the presence of a quinic acid. Co-elution with 3,5- and 4,5-O-dicaffeoylquinic acids previously isolated in our lab [36], enabled the identification of two of these peaks. In the case of 4,5-O-dicaffeoylquinic acid, the mass spectra were in accordance with the literature exhibiting one more fragment at *m/z* = 172.9 [quinic acid–H–H_2_O]^−^ which is diagnostic for the presence of 4-O-caffeoyl substituted quinic acid. This fragment is not observed in the other isomers [37].

### 3.2. Identification of Flavone and Flavonol Glycosides

Seventeen flavonoid (flavone and flavonol) glycosides were detected and identified in the chamomile extracts (Table 1). Flavones were represented mainly by apigenin derivatives which are considered as the active principles of *M. recutita*. Luteolin derivatives (luteolin and chrysoeriol glycosides) were also detected as minor compounds. Apigenin derivatives are characterized by UV absorbance, at ~330–337 nm (Βand I). Sugar substitution on position 7 does not influence the UV spectra while further esterification with acetic or malonic acid on the sugar has no effect on the UV absorbance [38]. On the other hand, sugar substitution on 4′ of the aglycone would cause a hypsochromic shift on Band I towards 325 nm. Based on these observations, compounds **13,** and **19–21** and **23** were assigned to apigenin-7-O-glycosides. Compound **13** was unambiguously assigned to apigenin-7-O-glucoside based on its fragmentation pattern and the use of a reference standard. Compounds **21** and **23** were identified as apigenin-7-acetylhexoside based on their fragmentation pattern and on literature reports. The loss of 204 amu (atomic mass unit) is attributed to the acetylated hexose and the lack of any intermediate fragments indicated that the acetyl group is placed on the sugar moiety. Different substitution sites of the acetyl group on the sugar give different compounds with different polarities and therefore retention times (compounds **21** and **23**). Similarly, compounds **19** and **20** were assigned to previously reported apigenin-7-O-acetylmalonyl-hexoside [39]. Their identification was based on MS spectra recorded in both the negative and positive ionization mode. Especially in the negative ionization mode, the fragments at *m/z* = 515.0 [M-CO_2_-H]^−^ indicated a neutral loss of a CO_2_ which is typical of malonyl groups. The more abundant compound **20**, was assigned to the more favorable apigenin-7-O-(6′′-malonyl)-glucoside, which is one of the main apigenin derivatives of chamomile. Concerning luteolin-7-O-glucoside (**8**) and chrysoeriol-7-O-hexoside (**14**) the addition of one extra oxygen substitution on C-3′ causes a bathochromic shift of band I at ~347 nm, whereas Band II exhibits a splitting of its peak which gives rise to a characteristic shoulder at 267 nm. Compound **8** was unambiguously identified by the use of a reference standard, while compound **14** is reported in the literature. In the chamomile extracts studied eight flavonol glycosides were detected and identified. To the best of our knowledge, this is the first time that so many flavonols are reported by the use of HPLC-PDA-MS in chamomile flowers. In principle, the presence of a hydroxyl group at position 3 of the flavonoid moiety causes a strong bathochromic shift of the Band I to 350–373 nm, but the exact shift depends greatly on the sugar substitution [38]. For example, a sugar group in position 3 causes an hypsochromic shift compared to the aglycone alone and the maximum absorbance appears at ~355 nm. This was the case of quercetagenin-3-O-glucoside (**3**), patuletin-3-O-glucoside (**7**), isorhamnetin-3-O-glucoside (**11**) and isorhamnetin-3-O-malonylhexoside (**16**). On the contrary, a 7-O- sugar substitution does not alter the UV spectra compared to the aglycone and so, Band I appears at ~370 nm. Therefore, compounds **5**, **6**, **10** and **18** were identified as quercetin-7-O-glucoside, patuletin-7-O-glucoside, isorhamnetin-7-O-glucoside and isorhamnetin-7-O-malonylhexoside, respectively. Furthermore, patuletin and quercetagenin derivatives exhibited a characteristic shift of Band II at 275 nm as shoulder, due to the oxygen substitution on C-6. This feature is of diagnostic importance to discriminate between the isobaric myricetin and quercetagenin. In all cases neutral losses (162 amu) were characteristic of the presence of sugar groups. Compounds **3**, **5**, **7** and **10** were unambiguously identified by co-elution with isolated compounds from *M. recutita* [34]. Finally, malonylhexoside derivatives of isorhamnetin (**16** and **18**) exhibited characteristic neutral losses of CO_2_ in their MS fragmentation patterns (Table 1).

### 3.3. Identification of Polyamine Conjugates

The last group of compounds eluting between 49.92 and 54.25 min were attributed to p-coumaroyl polyamines. Tetra-trans-p-coumaroyl thermospermine **29** was previously isolated in our lab from *M. pubescens* [34], and all the UV and MS data were identical to the lab isolate. Compound **28** had identical UV and MS spectral data but was slightly more polar and was assigned to the isobaric N1(E)-N5(E)-N10(E)-N14(E)- tetra-trans-p-coumaroyl spermine in accordance with the literature data. Introduction of cis-p-coumaroyl moieties on the spermine/thermospermine skeleton causes a shift of UV maximum to lower nanometers. Thus compound **27** (UV max. at 290 nm) was assigned to N1(Z)-N5(Z)-N9(Z)-N14(Z)- tetra-p-coumaroyl spermine/thermospermine (cis and trans-isomers), while compound **26** was attributed to N1(Z)-N5(Z)-N10(Z)-N14(Z)-tetra-p-cis-coumaroyl spermine/thermospermine having a maximum absorbance at 275 nm.

The main constituents in chamomile flowers, i.e., apigenin derivatives, flavonols and dicaffeoylquinic acid derivatives, were quantified (Table 2). The quantitation was carried out according to the European Pharmacopeia guidelines and the amounts of phenolic compounds were expressed as %*w/w* of dry herbal drug. For comparison purposes the content of the same phenolic compounds of three *M. recutita* varieties V3, V4 and V5 cultivated under the same conditions, was also estimated (Figure 1 and Figure 2, Table 2). As what concerns the Greek populations, the results showed that the total apigenin derivatives and the content of total caffeoylquinic acids were higher in the S2 population (0.39%, versus 0.31 %*w/w* in the S1 population and 0.96% versus 0.81 %*w/w* in the population S1, respectively). However, the amount of flavonols was higher in the S1 population (0.71 %*w/w*) as compared to the S2 (0.64 %*w/w*), (Table 2). The S2 population was comparable to the Banatska variety (V3), although somewhat better regarding the flavonol content. Both Greek populations had a richer content in phenolic constituents compared to Lutea and Goral varieties.

### 3.4. Total Phenolics, Total Flavonoid Content and Antioxidant Activity

**Figure 4:**
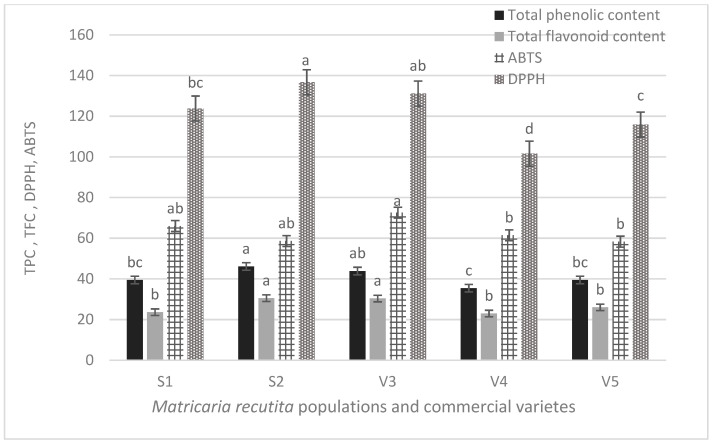
Total phenolic content (TPC, mg GAE/g extract), total flavonoid content (TFC, mg CATE/g extract) and antioxidant capacity (estimated by DPPH and ABTS assays, mg TE/g extract) of *Matricaria recutita* flowers; bars with the same color followed by the different letter were significantly different (α = 0.05).

The results of TPC, TFC and antioxidant activity, estimated by the DPPH and ABTS method, of S1, S2, V3, V4 and V5 chamomile extracts are presented in Figure 4. TPC ranged between 35.5 mg GAE/g extract in V4 variety and 46.15 mg GAE/g in S2 population, not statistically different from V3 variety. TFC was almost equal in S2 and V3 extracts (30.53 and 30.34 mg GAE/g, respectively), and the lowest; 22.96 mg GAE/g, was found in V4 variety. Concerning the antioxidant capacity, the highest ABTS was estimated in V3 variety (72.55 mg TE/g), and S2 and V5 exhibited the lowest one (58.55 and 58.27 mg TE/g respectively). Nevertheless, S2 showed the highest DPPH value (136.68 mg TE/g), followed by V3 (131.1 mg TE/g), and the lowest one was detected in V4 (101.57 mgTE/g).

## 4. Discussion

A thorough literature survey revealed that while there is a plethora of reports on the qualitative profile of *M. recutita* flos, there are only a few reports on the quantitative data. This is the first report on the phenolic constituents of Greek *M. recutita* flos, under cultivation conditions. Nováková et al. [40], reported first on the content of phenolics in commercial varieties, such as the Czech diploid variety Bohemia and the Slovak tetraploid variety Goral, all cultivated in Czech Republic. To the best of our knowledge, our work is the first report on the chemical content of the commercial varieties of Banatska and Lutea, as well as the first report on Goral variety from experimental cultivation in Greece. Unfortunately, there are many qualitative differences between our samples and the samples studied by Nováková et al. [40], to allow any comparison. Beside apigenin-7-glycoside and some flavonol glycosides, like rutin and quercitrin, they have determined flavonoid aglycons, which were not present in detectable amounts in our samples. Furthermore, they have determined chlorogenic acid, while dicaffeoylquinic acid derivatives are not detected as well. On the contrary, Goral variety cultivated in Greece seemed to be richer in phenolic derivatives, similarly to all other samples included in this study. Furthermore, in our study apigenin was not detected in the chamomile samples under investigation. This is possibly due to the appropriate drying conditions, since the herbal material was air dried in a dark, well-ventilated space. Enzymatic cleavage of glucose and releasing of aglycone were observed after harvesting in conditions without adequate air circulation [41]. The presence of dicaffeoylquinic acid derivatives was first mentioned by Mulinacci et al. [42], who detected both caffeic acid and ferulic acid derivatives in their methanolic chamomile extracts. In brief, they identified 5-caffeoylquinic acid, 3-caffeoylquinic acid, 4-caffeoylquinic acid, quinic acid, ferulic acid-1-O-glycoside, caffeoylquinic acid derivative, ferulic acid-7-O-glycoside, dicaffeoylquinic acid derivative, 1,3-dicaffeoylquinic acid. Caffeoylquinic acids were also measured in Greek populations and their content was almost similar to Banatska variety (0.96 %*w/w* and 0.93 %*w/w*, respectively), though it was lower in the varieties V4 and V5 (0.57—0.68 %*w/w*) (Table 2).

Concerning the flavonoid content there are many differences among chamomile samples in the literature. Of most importance is the study by Franke and Schilcher [43] who determined the flavonoid content in chamomile flowers from samples originating from 12 different locations and cultivated under the same conditions. It was found that the flavonoids ranged from 1.0% to 2.57%. However, no detailed data are provided regarding the type of flavonoids, as they were determined photometrically. Within this aspect, precise quantitative data on *M. recutita* flos were given quite recently by Avula [27]. They determined and quantified ten phenolic compounds in samples of *Matricaria* flowers of different origin, from several botanical gardens and commercial sources: cis and trans-2-β-D-glucopyranosyloxy-4-methoxycinnamic acid, chlorogenic acid, quercetagetin-7-β-D-glucopyranoside, apigenin-7-O-β-D-glucoside, apigenin-7-O-(6′′-O-acetyl-β-D-glucopyranoside), apigenin and en-yn-dicycloether. Nevertheless, no data are given concerning the rest of the flavonol derivatives and caffeoylquinic acids, whereas no data are provided regarding the chamomile varieties they studied.

The amount of total apigenin glycosides in the Greek populations are within the range of the Eur. Pharmacopoeia (minimum content 0.25 %*w/w*), therefore they are in compliance with the European regulations. Although apigenin and its derivatives are considered responsible for the bioactivity [19], the presence of other groups of flavonoids, phenolic acids and coumaroyl derivatives, contribute also to the health promoting properties of chamomile. In particular, patuletin has antioxidant and anti-inflammatory properties, and its potential as immunosuppressive and antiarthritic candidate has been shown [44,45]. Other studies have demonstrated that quercetin and luteolin could inhibit the accumulation of sorbitol in human erythrocytes, suggesting thus that chamomile beverages could contribute to the prevention of the progress of hyperglycemia and diabetic complications. Moreover, quercetin, quercetagetin and patuletin demonstrated antiproliferative activity in a dose-dependent manner [46]. On the other hand, it was demonstrated that isomers of tetra-p-coumaroyl thermospermines and tetra-p-coumaroyl spermines are promising antagonists, exerting positive effects on substance P/neurokinin-1 receptor-related diseases, including HER2- positive cancers, pain, mood disorders, and insomnia [47].

Concerning the total flavonols, as already mentioned, S2 population exhibited lower amounts than the S1, however both excelled compared to varieties V3, V4 and V5. It is also remarkable that although the variety V3—Banatska (diploid) was superior to the tetraploid ones when the total apigenin derivatives and the total caffeoylquinic acids are taken into consideration, regarding total flavonols the data differ. V3—Banatska and V4—Lutea exhibited the lower % yield in total flavonols (0.53 and 0.52 %*w/w*, respectively), though the other tetraploid variety V5—Goral gives higher total flavonols amount (0.57 %*w/w*) (Table 2). It should be noted that the phenolics % content in the tetraploid varieties ranged within similar limits, possibly due to their genetically similar origin [48]. Moreover, the content of bioactive compounds is attributed to several factors, such as the genotype, climatic conditions, agricultural practices, selection etc. The observed differences, among the commercial varieties and the populations in our study, when compared to the literature data, may be also due in some extent to the above-mentioned reasons, or the plant part (flowerheads, including tubular and ligulate flowers), the flowering stage during harvesting, the drying, or the extraction conditions.

S2 population contained an equal amount of apigenin derivatives (0.39 %*w/w*) as the diploid V3—Banatska (0.39 %*w/w*), while prevailed over the tetraploid ones (V4—Lutea, V5—Goral: 0.29 %*w/w* and 0.26 %*w/w* respectively) (Table 2). Svehlikova and Repčák [49] have shown that the percentage of apigenin glucoside and its acylated derivatives were higher in the ligulate florets and in the anthodia of diploid variety, comparing to the tetraploid, though the total apigenin quantity in the anthodium was higher in the tetraploid one. Faehnrich et al. [50] found that there was no significant difference in apigenin glycosides content between diploid (HUN2) and the tetraploid varieties Manzana and Lutea. Additionally, it has been shown that apigenin reaches its maximum content at full flowering, concluding that this stage is the most suitable, not only for the maximum of herbage yield, but also for the valuable active compounds [49]. The TPC values, estimated for the extracts of chamomile Greek populations and commercial varieties were in accordance and correlate well (data not shown) to the total amount of the identified caffeoylquinic acids (Table 2), which means that 3,5-dicaffeoylquinic acid and 4,5-dicaffeoylquinic acid contribute mainly to the total phenolic content.

The highest TFC values were recorded in S2 population and V3 variety and correlate (data not shown) with the corresponding total apigenin derivatives’ amount found by HPLC (Table 2), followed by V5 > S1 and V4. Nevertheless, according to the HPLC data, S1 population contained a similarly high amount of flavonoids (sum of total apigenin derivatives and total flavonols, Table 2), comparable to that of S2. A possible explanation for these differences might be, that the TFC method is selective for flavonols and for luteolin type flavones, therefore, apigenin derivatives are poorly measured [51].

Polyphenols are known for their antioxidant activity. Based on the results of Figure 4, S1, S2, V3, V4 and V5 chamomile extracts possess antioxidant capacity. In particular, radical scavenging activity measured against DPPH, was estimated in the following order in populations and varieties: S2 > V3 > S1 > V5 > V4, following the order of total polyphenols, as these were estimated in the extract by HPLC. However, the antioxidant activity of the extracts, measured by the ABTS method, was in slightly different order: V3 > S1 > V4 > S2 = V5. This difference is possibly explained by the different mode of the assessment of the antioxidant capacity, between DPPH and ABTS methods.

Despite the fact that chamomile (*M. recutita*) is a popular infusion, and known for its polyphenol content (flavonoids, phenolic acids, coumarins etc), data on its antioxidant activity are limited. Rusaczonek et al. [52] reported on the antioxidant activity of some popular infusions (*Camellia sinensis* and herbal teas). Chamomile infusion demonstrated the lowest TFC and antioxidant activity, compared to Green Tea, Black Tea, White Tea, and Lemon Balm and Peppermint infusion as well. Moreover, values of antioxidant activity, e.g., DPPH, are presented in the literature by different methods, and the results, consequently, are expressed differently [53,54]. Additionally, the content of polyphenols–which are associated to the antioxidant activity of plants, is affected by the genotype, environmental factors, cultivation practices etc.

Alibabaei et al. [55] found that chamomile 70% ethanolic extract exhibited free radical scavenging activity (IC_50_ value 50 μg/mL), that is possibly attributed to the memory enhancing activity. In the same study, the total phenolics and flavonoids were estimated at 78.4 mg gallic acid equivalent per/g dried extract, and 47.6 mg rutin equivalent/g respectively. Mierina et al. [56], reported that 96% ethanol chamomile extract was the most effective concerning the highest free radical scavenging activity, though they did not find any correlation among TPC and free radical scavenging activity. 

The results of our study showed that the extracts of chamomile populations and varieties under investigation, exhibited antioxidant activity, and the highest one was recorded in the diploid variety and Greek populations. This is of importance, since our aim was to identify the best chamomile population in terms of phenol content and antioxidant potential, suitable for cultivation and furthermore for development of dietary products.

## 5. Conclusions

In the present work, the qualitative and quantitative poly polyphenolic profile of Greek chamomile was assessed for the first time. In addition, three commercial varieties of *M. recutita* cultivated under the same conditions were also evaluated for comparison reasons. To the best of our knowledge, this is the first detailed report on the various flavonoids and phenolics present in the Greek chamomile populations and in well-established commercial varieties. Results demonstrate that Greek chamomile populations were particularly rich in phenolic content, and their extracts exert antioxidant activity, rendering them a promising material, with desirable characteristics for medicinal uses and industrial applications as food supplement.

## Figures and Tables

**Table 1:** MS fragmentation and UV-vis absorption data of the compounds detected in the methanol extract of *Matricaria recutita* flowers of the studied samples.

	Rt (min)	UV (nm)	*m/z* (-) Negative Mode	*m/z* (+) Positive Mode	Identification	Mode of Identification
**1**	4.17	296, 326	191.1 [quinic acid-H]^−^, 352.9 [M-H]^−^	163.0 [caffeic-H_2_O+H]^+^, 354.8 [M+H]^+^	chlorogenic acid	UV/MS, std
**2**	4.37	279, 301	134.1, 149.1, 193.1, 355.1 [M-H]^−^, 711.1 [2M-H]^−^	177.2, 195.1, 378.8 [M+Na]^+^	cis-2-hydroxy-4-methoxycinnamic-oxo-2-O-β-D-glucopyranoside	UV/MS, std, NMR
**3**	7.88	259, 275sh, 354	317.0 [A-H]^−^, 478.9 [M-H]^−^	318.9 [A+H]^+^, 481.0 [M+Na]^-^	quercetagenin-3-O-glucoside	UV/MS, std, NMR
**4**	7.95	295, 318	134.1, 148.9, 193.0, 354.9 [M-H]^−^, 711.0 [2M-H]^−^	379.0 [M+Na]^+^	trans-2-hydroxy-4-methoxycinnamic-oxo-2-O-β-D-glucopyranoside	UV/MS, std, NMR
**5**	11.62	255, 270sh, 370	300.9 [A-H]^−^, 463.1 [M-H]^−^	303.0 [A+H]^+^, 464.7 [M+H]^+^	quercetin-7-O-glucoside	UV/MS, std, NMR
**6**	13.07	258, 274sh, 369	330.9 [A-H]^−^, 492.8 [M-H]^−^	333.0 [A+H]^+^, 495.1 [M+H]^+^	patuletin-7-O-glucoside	UV/MS, ref
**7**	13.34	259, 275sh, 356	331.0 [A-H]^−^, 493.0 [M-H]^−^	332.9 [A+H]^+^, 495.1 [M+H]^+^	patuletin-3-O-glucoside	UV/MS, std, NMR
**8**	14.48	255, 267sh, 347	284.9 [A-H]^−^, 446.9 [M-H]^−^	287.1 [A+H]^+^, 449.1 [M+H]^+^	luteolin-7-O-glucoside	UV/MS, std
**9**	16.37	296, 325	173.1, 178.9, 191.1, 514.8 [M-H]^−^	516.8 [M+H]^+^	dicaffeoylquinic acid derivative	UV/MS
**10**	17.85	254, 270sh, 370	314.9 [A-H]^−^, 477.1 [M-H]^−^	316.9 [A+H]^+^, 479.0 [M+H]^+^	isorhamnetin-7-O-glucoside	UV/MS, NMR
**11**	18.23	254, 269sh, 352	313.8 [A-2H]^−^, 315.0 [A-H]^−^, 476.9 [M-H]^−^	316.9 [A+H]^+^	isorhamnetin-3-O-glucoside	UV/MS, std
**12**	18.70	298, 327	179.1 [caffeic acid-H]^−^, 191.1 [quinic acid-H]^−^, 352.9 [M-caffeoyl-H]^−^, 514.9 [Μ-H]^−^	539.0 [M+H]^+^	3,5-O-dicaffeoylquinic acid	UV/MS, std
**13**	19.53	267, 336	268 [A-2H]^−^, 269.1 [A-H]^−^, 431.0 [M-H]^−^	271.1 [A+H]^+^, 433.1 [M+H]^+^	apigenin-7-O-glucoside	NMR/UV/MS, std
**14**	20.53	252, 266sh, 347	298.7 [A-H]^−^, 445.9 [Μ-CH_3_-H]^−^, 460.9 [Μ-H]^−^	301.0 [A+H]^+^, 463.0 [M+H]^+^	chrysoeriol-7-O-glucoside	UV/MS, ref
**15**	21.03	298, 327	172.9, 179.0 [caffeic acid-H]^−^, 191 [quinic acid-H]^−^, 353.0 [Μ-caffeoyl-H]^−^, 515.1 [Μ-H]^−^	162.3 [caffeic acid-H_2_O +H]^+^	4,5-O-dicaffeoylquinic acid	UV/MS, std
**16**	22.64	255, 267sh, 354	314.9 [A-H]^−^	316.9 [A+H]^+^, 565.0 [M+H]^+^, 586.9 [M+Na]^+^	isorhamnetin-3-O-malonylhexoside tentatively	UV/MS
**17**	23.17	300, 319	160.8, 193.1, 517.3 [M-H]^−^	163.0, 540.9 [M+Na]^+^	acylated derivative of cis-2-hydroxy-4-methoxycinnamic-oxo-2-O-β-D-glucopyranoside	UV/MS
**18**	25.66	254, 268sh, 367	315.1 [A-H]^−^	317.1 [A+H]^+^, 565.2 [M+H]^+^	isorhamnetin-7-O-malonylhexoside tentatively	UV/MS
**19**	27.40	266, 330	269.1 [A-H]^−^	271.1 [A+H]^+^, 519.1 [M+H]^+^	apigenin-7-O-malonylhexoside	UV/MS
**20**	28.21	267, 336	269.0 [A-H]^−^, 472.9 [M-CO_2_-H]^−^	271.1 [A+H]^+^, 519.1 [M+H]^+^	apigenin-7-O-(6′′-malonyl)-glucoside	UV/MS, ref
**21**	29.74	267, 336	269.2 [A-H]^−^, 473.0 [M-H]^−^	271.1 [A+H]^+^, 474.6 [M+H]^+^	apigenin-7-acetylhexoside	UV/MS, ref
**22**	30.62	298, 324	160.9, 193.1, 517.0 [M-H]^−^	-	acylated derivative of trans-2-hydroxy-4-methoxycinnamic-oxo-2-O-β-D-glucopyranoside	UV/MS
**23**	36.28	267, 336	269.0 [A-H]^−^, 473.0 [M-H]^−^	271.1 [A+H]^+^, 475.4 [M+H]^+^	apigenin-7-acetylhexoside isomer	UV/MS, ref
**24**	37.86	267, 336	269.0 [A-H]^−^, 515.0 [M-CO_2_-H]^−^	271.1 [A+H]^+^, 561.1 [M+H]^+^	apigenin-7-O-acetylmalonyl-hexoside	UV/MS, ref
**25**	38.99	267, 334	268.9 [A-H]^−^, 515.2 [M-CO_2_-H]^−^	271.1 [A+H]^+^, 560.9 [M+H]^+^	apigenin-7-O-acetylmalonyl-hexoside	UV/MS, ref
**26**	49.92	275	545.0, 665.1, 785.3 [M-H]^−^	147.1 [coumaroyl+H]^+^, 641.3, 787.3 [M+H]^+^	N1(Z)-N5(Z)-N10(Z)-N14(Z)-tetra-p-coumaroyl spermine/thermospermine (cis-isomers)	UV/MS, ref
**27**	51.13	290	545.5, 665.1, 785.3 [M-H]^−^	147.2 [coumaroyl+H]^+^, 641.2, 787.4 [M+H]^+^	N1(Z)-N5(Z)-N9(Z)-N14(Z)- tetra-p-coumaroyl spermine/thermospermine (cis and trans-isomers)	UV/MS, ref
**28**	52.01	292, 308	544.9, 665.1, 785.3 [M-H]^−^	146.9 [coumaroyl+H]^+^, 641.3, 787.4 [M+H]^+^	N1(E)-N5(E)-N10(E)-N14(E)- tetra-p-coumaroyl spermine (trans-isomer)	UV/MS, ref
**29**	54.25	297, 308	545.1, 665.1, 785.3 [M-H]^−^	147.1 [coumaroyl+H]^+^, 495.2 [M-2 x coumaroyl+H]^+^, 641.3 [M-coumaroyl+H]^+^, 787.5 [M+H]^+^, 809.3 [M+Na]^+^	N1(E)-N5(E)-N9(E)-N14(E)- tetra-p-coumaroyl thermospermine (trans-isomer)	UV/MS, ref

A: aglycon; std standard

**Table 2:** Amounts (%*w/w* dry weight of herbal drug) of apigenin derivatives, flavonols and dicaffeoylquinic acid derivatives, in *Matricaria recutita* flowers (*r* = 3).

	S1	S2	V3-Banatska	V4-Lutea	V5-Goral
Apigenin-7-O-glucoside	0.147 ± 0.018 ^a^	0.17 ± 0.02 ^a^	0.17 ± 0.02 ^a^	0.10 ± 0.02 ^b^	0.10 ± 0.01 ^b^
Apigenin-7-O-acetylglucoside	0.108 ± 0.007 ^b^	0.15 ± 0.01 ^a^	0.15 ± 0.01 ^a^	0.08 ± 0.01 ^c^	0.07 ± 0.01 ^d^
Apigenin-7-O-malonylacetylglucoside	0.052 ± 0.002 ^c^	0.07 ± 0.01 ^b^	0.08 ± 0.01 ^b^	0.10 ± 0.01 ^a^	0.09 ± 0.01 ^a^
**Total apigenin derivatives**	**0.31 ± 0.03 ^b^**	**0.39 ± 0.02 ^a^**	**0.40 ± 0.03 ^a^**	**0.28 ± 0.01 ^bc^**	**0.26 ± 0.02 ^c^**
Patuletin-3-O-glucoside	0.49 ± 0.02 ^a^	0.45 ± 0.01 ^b^	0.22 ± 0.025 ^e^	0.25 ± 0.01 ^d^	0.30 ± 0.01 ^c^
Patuletin-7-O-glucoside	0.10 ± 0.01 ^d^	0.10 ± 0.01 ^d^	0.23± 0.01 ^a^	0.20 ± 0.01 ^c^	0.21 ± 0.01 ^b^
Quercetagenin-3-O-glucoside	0.11 ± 0.01 ^a^	0.09 ± 0.01 ^b^	0.08 ± 0.01 ^c^	0.06 ± 0.01 ^d^	0.06 ± 0.01 ^d^
**Total flavonols**	**0.71 ± 0.02 ^a^**	**0.64 ± 0.01 ^b^**	**0.53 ± 0.02 ^d^**	**0.51 ± 0.01 ^d^**	**0.57 ± 0.03 ^c^**
3,5-dicaffeoylquinic acid	0.58 ± 0.04 ^c^	0.70 ± 0.02 ^a^	0.63 ± 0.03 ^b^	0.29 ± 0.01 ^e^	0.36 ± 0.01 ^d^
4,5-dicaffeoylquinic acid	0.23 ± 0.02 ^d^	0.26 ± 0.01 ^c^	0.30 ± 0.01 ^ab^	0.29 ± 0.01 ^b^	0.32 ± 0.01 ^a^
**Total caffeoylquinic acids**	**0.81 ± 0.04 ^b^**	**0.96 ± 0.02 ^a^**	**0.93 ± 0.03 ^a^**	**0.58 ± 0.01 ^d^**	**0.68 ± 0.01 ^c^**

The data represent the mean values ± standard deviation. Values with different lowercase letter within the same line are statistically significant different (α = 0.05).

## Supplementary Materials

The following are available online at https://www.mdpi.com/article/10.3390/foods10102345/s1, Figure S1: HPLC-UV (330 nm) chromatogram of polar extracts from the two Greek chamomile populations; Figure S2: HPLC-UV (330 nm) chromatogram of polar extracts from chamomile populations under investigation: Banatska variety (V3), Lutea variety (V4), Goral variety (V5) and S1 and S2 Greek populations.
