# Supplementary material for: Phenolic Profile by HPLC-PDA-MS of Greek Chamomile Populations and Commercial Varieties and Their Antioxidant Activity
Source: Foods. 2021 Oct 1;10(10):2345. doi: 10.3390/foods10102345 (PMC8535277; doi:10.3390/foods10102345)
Supplement: Supplementary file 1 [file foods-10-02345-s001.zip › foods-1395277-supplementary.pdf]

Phenolic Profile by HPLC-PDA-MS of Greek Chamomile Populations and Commercial Varieties and Their Antioxidant Activity

Nektaria Tsivelika[1,2], Maria Irakli[2], Athanasios Mavromatis[1], Paschalina Chatzopoulou[2] and Anastasia Karioti[3]

[1]*Laboratory of Genetics and Plant Breeding, Department of Agriculture, Faculty of Agriculture Forestry and Natural Environment, Aristotle University of Thessaloniki, Thessaloniki, Greece*

[2]*Hellenic Agricultural Organization – DEMETER, Institute of Breeding and Plant Genetic Resources, Thermi, 57001, Greece*

[3]*Laboratory of Pharmacognosy, School of Pharmacy, Aristotle University of Thessaloniki, University Campus, 54124, Thessaloniki, Greece*

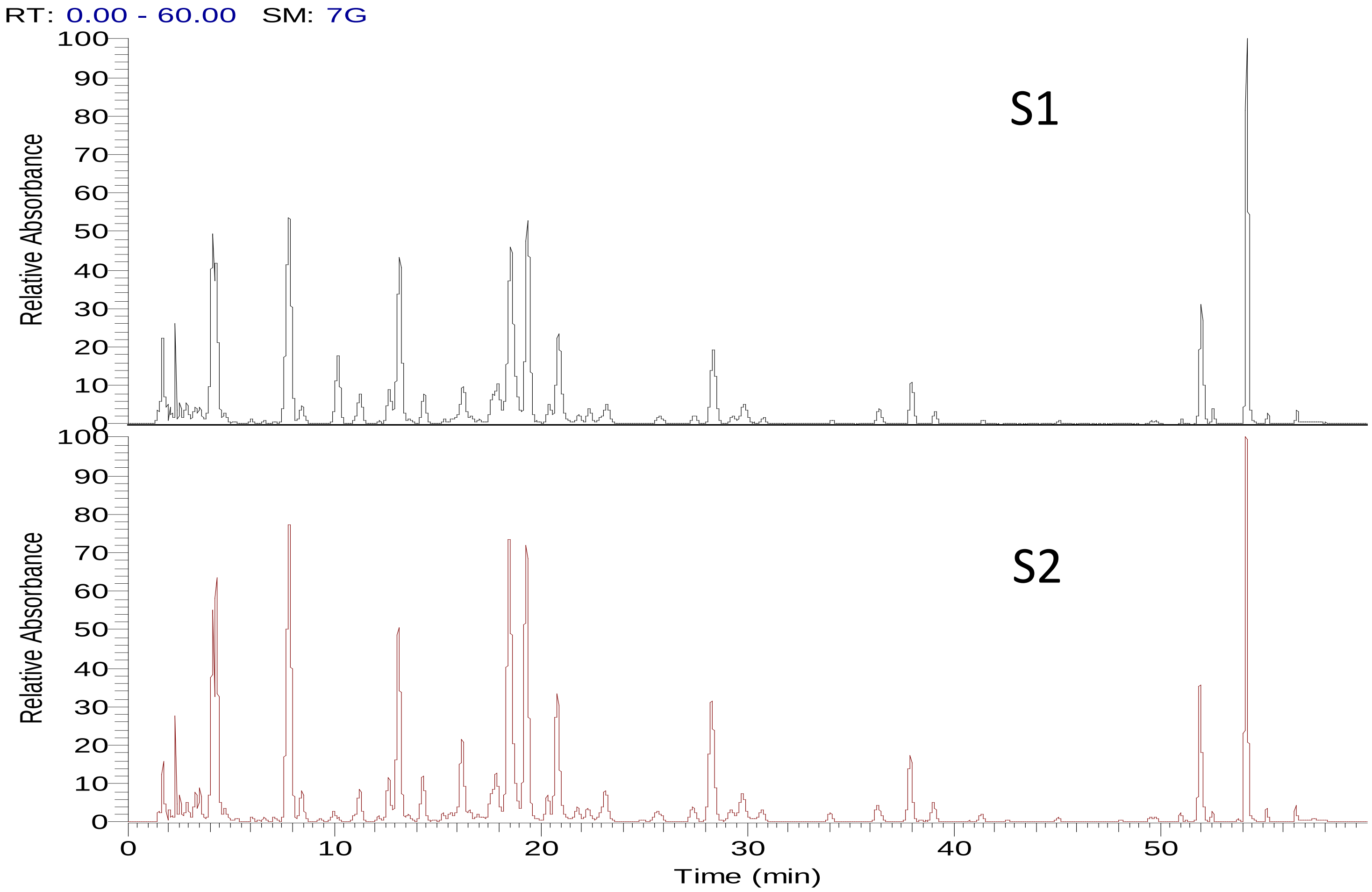

Figure S1. HPLC-UV (330 nm) chromatogram of polar extracts from the two Greek chamomile populations, S1 and S2.

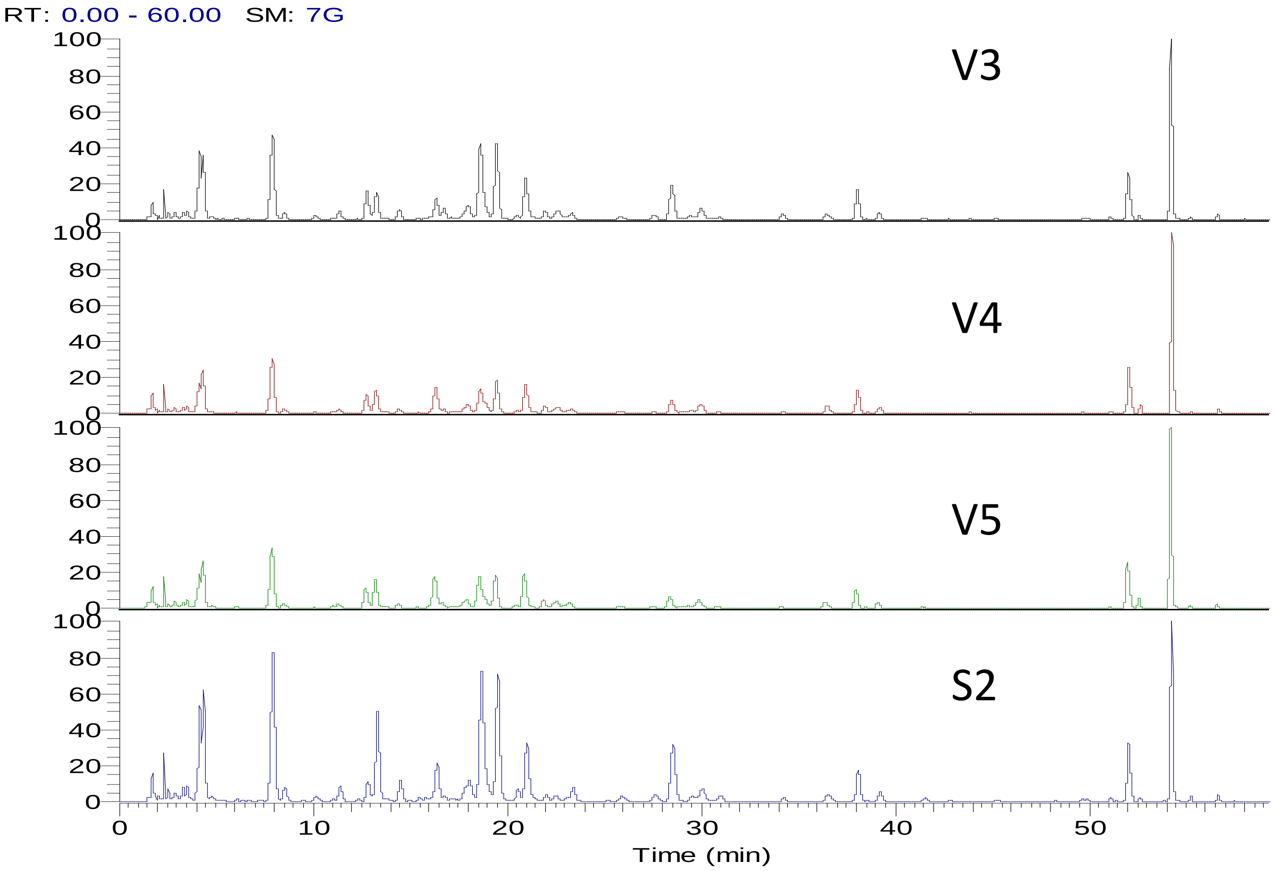

Figure S2. HPLC-UV (330 nm) chromatogram of polar extracts from chamomile populations under investigation: Banatska variety (V3), Lutea variety (V4), Goral variety (V5) and the S2 Greek population.
